# Triclinic modification of di-*n*-butyl­bis(2-hydroxy­benzoato-*κ* ^2^ *O* ^1^,*O* ^1′^)tin(IV)

**DOI:** 10.1107/S1600536808023799

**Published:** 2008-08-20

**Authors:** Reza Reisi, Misni Misran, Kong Mun Lo, Seik Weng Ng

**Affiliations:** a Department of Chemistry, University of Malaya, 50603 Kuala Lumpur, Malaysia

## Abstract

The Sn atom in the title compound, [Sn(C_4_H_9_)_2_(C_7_H_5_O_3_)_2_], is chelated by the carboxyl­ate groups of 2-hydroxy­benzoate liagnds, and exists in a six-coordinate skew-trapezoidal bipyramidal coordination geometry [C—Sn—C = 140.1 (3)°].

## Related literature

For the monoclinic modification, see: Narula *et al.* (1992 ▶). For a review of the structural chemistry of organotin carboxyl­ates, see: Tiekink (1991 ▶, 1994 ▶). For a discussion of skew-trapezoidal bipyramidal diorganotin bis­(chelates), see: Ng *et al.* (1987 ▶).
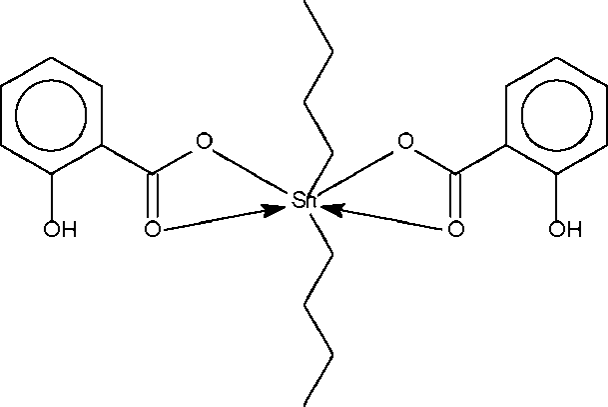


## Experimental

### 

#### Crystal data

[Sn(C_4_H_9_)_2_(C_7_H_5_O_3_)_2_]
*M* *_r_* = 507.13
Triclinic, 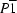

*a* = 9.1652 (2) Å
*b* = 11.2111 (2) Å
*c* = 12.2620 (2) Å
α = 94.759 (1)°
β = 106.872 (1)°
γ = 108.586 (1)°
*V* = 1121.24 (4) Å^3^
*Z* = 2
Mo *K*α radiation
μ = 1.17 mm^−1^
*T* = 100 (2) K
0.25 × 0.20 × 0.15 mm

#### Data collection

Bruker SMART APEX diffractometer
Absorption correction: multi-scan (*SADABS*; Sheldrick, 1996 ▶) *T*_min_ = 0.758, *T*_max_ = 0.844
11666 measured reflections
5068 independent reflections
4633 reflections with *I* > 2σ(*I*)
*R*_int_ = 0.034

#### Refinement

*R*[*F* ^2^ > 2σ(*F* ^2^)] = 0.059
*wR*(*F* ^2^) = 0.189
*S* = 1.18
5068 reflections
262 parameters
2 restraints
H-atom parameters constrained
Δρ_max_ = 2.57 e Å^−3^
Δρ_min_ = −1.40 e Å^−3^

### 

Data collection: *APEX2* (Bruker, 2007 ▶); cell refinement: *SAINT* (Bruker, 2007 ▶); data reduction: *SAINT*; program(s) used to solve structure: *SHELXS97* (Sheldrick, 2008 ▶); program(s) used to refine structure: *SHELXL97* (Sheldrick, 2008 ▶); molecular graphics: *X-SEED* (Barbour, 2001 ▶); software used to prepare material for publication: *publCIF* (Westrip, 2008 ▶).

## Supplementary Material

Crystal structure: contains datablocks global, I. DOI: 10.1107/S1600536808023799/tk2286sup1.cif

Structure factors: contains datablocks I. DOI: 10.1107/S1600536808023799/tk2286Isup2.hkl

Additional supplementary materials:  crystallographic information; 3D view; checkCIF report

## Figures and Tables

**Table 1:** Hydrogen-bond geometry (Å, °)

*D*—H⋯*A*	*D*—H	H⋯*A*	*D*⋯*A*	*D*—H⋯*A*
O3—H3o⋯O2	0.84	1.96	2.599 (9)	132
O6—H6o⋯O5	0.84	2.00	2.626 (8)	131

**Fig. 1.**
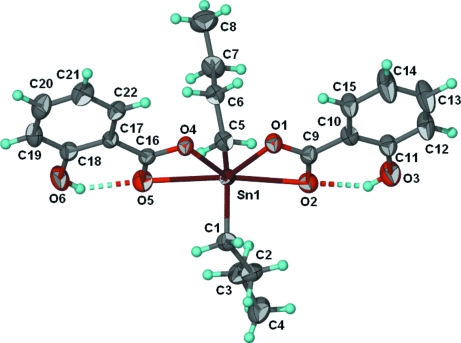
Thermal ellipsoid plot (Barbour, 2001) plot of the triclinic form of [Sn(C4H9)2(C7H5O3] at the 50% probability level. Hydrogen atoms are drawn as spheres of arbitrary radius.

## supplementary crystallographic information

### Comment

Diorganotin dicarboxylates generally exist as monomeric molecules in which the carboxylate groups chelate in an anisobidentate manner (Tiekink, 1991; 1994). The *R*_2_Sn unit is bent, and the geometry at tin is described as being skew-trapezoidal bipyramidal (Ng *et al.*, 1987). The title compound has been reported in a monoclinic form (Narula *et al.*, 1992). This structure has one *n*-butyl group in a *W* conformation and the other in a *U* conformation. In the present triclinic modification (Scheme I, Fig. 1), both groups adopt a *W* conformation. Intramolecular O-H···O hydrogen bonds are noted (Table 1).

### Experimental

Dibutyltin oxide (2 g, 8 mmol) and salicylic acid (2.2 g, 16 mmol) were heated in toluene (100 ml) in a Dean-Stark water apparatus. Slow evaporation of the filtered solution yielded colorless crystals.

### Refinement

Carbon-bound H-atoms were placed in positions (C–H 0.95 to 0.99 Å) and were included in the refinement in the riding model approximation, with *U*(H) set to 1.2–1.5*U*_eq_(C). The hydroxy H-atoms were similarly constrained (O–H 0.84 Å) but the hybridization of the oxygen atoms was assumed to be *sp*^2^.

The final difference Fourier map had a peak of 2.57 e Å^-3^ at 1.5 Å from the O5 and O6 atoms, and a deep hole of -1.40 e Å^-3^ at 1.5 Å from the H12 atom.

### Crystal data

**Table:**

[Sn(C_4_H_9_)_2_(C_7_H_5_O_3_)_2_]	*Z* = 2
*M**_r_* = 507.13	*F*_000_ = 516
Triclinic, *P*1	*D*_x_ = 1.502 Mg m^−^^3^
Hall symbol: -P 1	Mo *K*α radiation λ = 0.71073 Å
*a* = 9.1652 (2) Å	Cell parameters from 6302 reflections
*b* = 11.2111 (2) Å	θ = 2.5–27.7º
*c* = 12.2620 (2) Å	µ = 1.17 mm^−^^1^
α = 94.759 (1)º	*T* = 100 (2) K
β = 106.872 (1)º	Block, colorless
γ = 108.586 (1)º	0.25 × 0.20 × 0.15 mm
*V* = 1121.24 (4) Å^3^	

### Data collection

**Table:**

Bruker SMART APEX diffractometer	5068 independent reflections
Radiation source: fine-focus sealed tube	4633 reflections with *I* > 2σ(*I*)
Monochromator: graphite	*R*_int_ = 0.034
*T* = 100(2) K	θ_max_ = 27.5º
ω scans	θ_min_ = 1.8º
Absorption correction: Multi-scan(SADABS; Sheldrick, 1996)	*h* = −11→7
*T*_min_ = 0.758, *T*_max_ = 0.844	*k* = −14→14
11666 measured reflections	*l* = −15→15

### Refinement

**Table:**

Refinement on *F*^2^	Secondary atom site location: difference Fourier map
Least-squares matrix: full	Hydrogen site location: inferred from neighbouring sites
*R*[*F*^2^ > 2σ(*F*^2^)] = 0.059	H-atom parameters constrained
*wR*(*F*^2^) = 0.189	*w* = 1/[σ^2^(*F*_o_^2^) + (0.0604*P*)^2^ + 8.6498*P*] where *P* = (*F*_o_^2^ + 2*F*_c_^2^)/3
*S* = 1.18	(Δ/σ)_max_ = 0.001
5068 reflections	Δρ_max_ = 2.57 e Å^−^^3^
262 parameters	Δρ_min_ = −1.40 e Å^−^^3^
2 restraints	Extinction correction: none
Primary atom site location: structure-invariant direct methods	

### Fractional atomic coordinates and isotropic or equivalent isotropic displacement parameters (Å^2^)

**Table:**

	*x*	*y*	*z*	*U*_iso_*/*U*_eq_	
Sn1	0.50157 (6)	0.34322 (5)	0.64132 (4)	0.03511 (17)	
O1	0.6039 (7)	0.5445 (5)	0.6922 (5)	0.0424 (11)	
O2	0.4303 (7)	0.4748 (5)	0.7850 (5)	0.0495 (13)	
O3	0.4148 (9)	0.6313 (7)	0.9462 (6)	0.0687 (19)	
H3O	0.3802	0.5549	0.9111	0.103*	
O4	0.6470 (6)	0.3786 (5)	0.5355 (4)	0.0391 (11)	
O5	0.5384 (8)	0.1687 (6)	0.5037 (5)	0.0535 (14)	
O6	0.5995 (9)	0.0298 (5)	0.3515 (6)	0.0634 (18)	
H6O	0.5475	0.0276	0.3980	0.095*	
C1	0.2603 (9)	0.2912 (8)	0.5242 (7)	0.0452 (17)	
H1A	0.2522	0.3652	0.4873	0.054*	
H1B	0.2412	0.2201	0.4620	0.054*	
C2	0.1264 (10)	0.2505 (8)	0.5755 (8)	0.0512 (19)	
H2A	0.1354	0.3253	0.6296	0.061*	
H2B	0.0198	0.2243	0.5123	0.061*	
C3	0.1281 (12)	0.1422 (9)	0.6398 (9)	0.059 (2)	
H3A	0.2243	0.1738	0.7118	0.071*	
H3B	0.1410	0.0738	0.5911	0.071*	
C4	−0.0260 (15)	0.0841 (11)	0.6719 (11)	0.077 (3)	
H4A	−0.0159	0.0150	0.7140	0.116*	
H4B	−0.1217	0.0496	0.6009	0.116*	
H4C	−0.0389	0.1507	0.7212	0.116*	
C5	0.6353 (10)	0.2839 (7)	0.7842 (7)	0.0429 (16)	
H5A	0.6076	0.3066	0.8534	0.051*	
H5B	0.6034	0.1895	0.7669	0.051*	
C6	0.8165 (10)	0.3455 (7)	0.8106 (7)	0.0423 (16)	
H6A	0.8473	0.4399	0.8259	0.051*	
H6B	0.8436	0.3215	0.7415	0.051*	
C7	0.9178 (12)	0.3068 (9)	0.9151 (8)	0.059 (2)	
H7A	0.8917	0.3318	0.9845	0.070*	
H7B	0.8861	0.2124	0.9003	0.070*	
C8	1.0967 (13)	0.3666 (13)	0.9401 (10)	0.079 (3)	
H8A	1.1547	0.3411	1.0093	0.118*	
H8B	1.1288	0.4602	0.9538	0.118*	
H8C	1.1245	0.3381	0.8736	0.118*	
C9	0.5369 (9)	0.5664 (7)	0.7668 (6)	0.0391 (15)	
C10	0.5867 (10)	0.6976 (7)	0.8301 (6)	0.0391 (15)	
C11	0.5246 (11)	0.7235 (8)	0.9170 (7)	0.0466 (18)	
C12	0.5768 (15)	0.8474 (9)	0.9784 (8)	0.066 (3)	
H12	0.5344	0.8648	1.0375	0.079*	
C13	0.6896 (18)	0.9447 (9)	0.9535 (11)	0.085 (4)	
H13	0.7237	1.0298	0.9949	0.102*	
C14	0.7559 (19)	0.9206 (9)	0.8676 (11)	0.091 (5)	
H14	0.8361	0.9884	0.8522	0.109*	
C15	0.7029 (12)	0.7971 (8)	0.8060 (8)	0.053 (2)	
H15	0.7457	0.7799	0.7471	0.064*	
C16	0.6292 (8)	0.2670 (7)	0.4830 (6)	0.0350 (14)	
C17	0.7181 (8)	0.2616 (7)	0.4013 (6)	0.0344 (14)	
C18	0.6975 (10)	0.1431 (7)	0.3404 (7)	0.0420 (16)	
C19	0.7814 (12)	0.1408 (9)	0.2613 (7)	0.052 (2)	
H19	0.7649	0.0615	0.2163	0.063*	
C20	0.8863 (13)	0.2521 (10)	0.2488 (8)	0.061 (2)	
H20	0.9441	0.2489	0.1964	0.074*	
C21	0.9105 (12)	0.3695 (9)	0.3108 (8)	0.056 (2)	
H21	0.9849	0.4463	0.3020	0.067*	
C22	0.8242 (9)	0.3729 (7)	0.3858 (7)	0.0419 (16)	
H22	0.8380	0.4532	0.4276	0.050*	

### Atomic displacement parameters (Å^2^)

**Table:**

	*U*^11^	*U*^22^	*U*^33^	*U*^12^	*U*^13^	*U*^23^
Sn1	0.0326 (3)	0.0370 (3)	0.0356 (3)	0.00980 (19)	0.01499 (19)	0.00458 (18)
O1	0.048 (3)	0.040 (3)	0.042 (3)	0.015 (2)	0.020 (2)	0.006 (2)
O2	0.049 (3)	0.045 (3)	0.052 (3)	0.010 (2)	0.021 (3)	0.006 (2)
O3	0.078 (5)	0.059 (4)	0.071 (4)	0.009 (3)	0.048 (4)	0.003 (3)
O4	0.044 (3)	0.038 (3)	0.041 (3)	0.014 (2)	0.022 (2)	0.006 (2)
O5	0.058 (4)	0.046 (3)	0.060 (4)	0.010 (3)	0.035 (3)	0.010 (3)
O6	0.076 (4)	0.036 (3)	0.076 (4)	0.006 (3)	0.041 (4)	−0.002 (3)
C1	0.041 (4)	0.054 (4)	0.039 (4)	0.014 (3)	0.013 (3)	0.011 (3)
C2	0.040 (4)	0.051 (5)	0.065 (5)	0.017 (4)	0.020 (4)	0.019 (4)
C3	0.055 (5)	0.055 (5)	0.077 (6)	0.019 (4)	0.034 (5)	0.022 (5)
C4	0.080 (7)	0.070 (7)	0.098 (8)	0.019 (6)	0.062 (7)	0.014 (6)
C5	0.050 (4)	0.040 (4)	0.039 (4)	0.012 (3)	0.019 (3)	0.010 (3)
C6	0.048 (4)	0.039 (4)	0.041 (4)	0.013 (3)	0.017 (3)	0.010 (3)
C7	0.063 (6)	0.057 (5)	0.054 (5)	0.024 (4)	0.011 (4)	0.016 (4)
C8	0.056 (6)	0.104 (9)	0.074 (7)	0.037 (6)	0.008 (5)	0.020 (6)
C9	0.042 (4)	0.046 (4)	0.034 (3)	0.021 (3)	0.014 (3)	0.008 (3)
C10	0.049 (4)	0.036 (3)	0.032 (3)	0.017 (3)	0.011 (3)	0.009 (3)
C11	0.057 (5)	0.044 (4)	0.044 (4)	0.017 (4)	0.024 (4)	0.008 (3)
C12	0.099 (8)	0.055 (5)	0.054 (5)	0.027 (5)	0.043 (5)	0.003 (4)
C13	0.136 (11)	0.037 (5)	0.086 (8)	0.010 (6)	0.068 (8)	−0.001 (5)
C14	0.145 (12)	0.038 (5)	0.100 (9)	0.007 (6)	0.089 (9)	0.002 (5)
C15	0.073 (6)	0.044 (4)	0.051 (5)	0.017 (4)	0.038 (4)	0.009 (4)
C16	0.033 (3)	0.040 (4)	0.033 (3)	0.013 (3)	0.012 (3)	0.007 (3)
C17	0.036 (3)	0.041 (4)	0.031 (3)	0.017 (3)	0.013 (3)	0.008 (3)
C18	0.043 (4)	0.039 (4)	0.046 (4)	0.013 (3)	0.020 (3)	0.008 (3)
C19	0.068 (6)	0.055 (5)	0.041 (4)	0.026 (4)	0.025 (4)	0.003 (3)
C20	0.074 (6)	0.082 (7)	0.048 (5)	0.039 (5)	0.036 (5)	0.018 (4)
C21	0.064 (6)	0.059 (5)	0.060 (5)	0.023 (4)	0.038 (5)	0.028 (4)
C22	0.044 (4)	0.043 (4)	0.048 (4)	0.020 (3)	0.022 (3)	0.016 (3)

### Geometric parameters (Å, °)

**Table:**

Sn1—C1	2.118 (8)	C6—H6A	0.9900
Sn1—C5	2.117 (8)	C6—H6B	0.9900
Sn1—O1	2.106 (5)	C7—C8	1.485 (15)
Sn1—O2	2.561 (6)	C7—H7A	0.9900
Sn1—O4	2.090 (5)	C7—H7B	0.9900
Sn1—O5	2.645 (6)	C8—H8A	0.9800
O1—C9	1.288 (9)	C8—H8B	0.9800
O2—C9	1.259 (9)	C8—H8C	0.9800
O3—C11	1.348 (10)	C9—C10	1.467 (10)
O3—H3O	0.8400	C10—C11	1.396 (11)
O4—C16	1.296 (8)	C10—C15	1.395 (11)
O5—C16	1.247 (9)	C11—C12	1.386 (12)
O6—C18	1.346 (9)	C12—C13	1.368 (15)
O6—H6O	0.8400	C12—H12	0.9500
C1—C2	1.501 (11)	C13—C14	1.408 (15)
C1—H1A	0.9900	C13—H13	0.9500
C1—H1B	0.9900	C14—C15	1.382 (12)
C2—C3	1.503 (12)	C14—H14	0.9500
C2—H2A	0.9900	C15—H15	0.9500
C2—H2B	0.9900	C16—C17	1.472 (9)
C3—C4	1.534 (13)	C17—C22	1.383 (10)
C3—H3A	0.9900	C17—C18	1.397 (10)
C3—H3B	0.9900	C18—C19	1.405 (11)
C4—H4A	0.9800	C19—C20	1.362 (14)
C4—H4B	0.9800	C19—H19	0.9500
C4—H4C	0.9800	C20—C21	1.382 (14)
C5—C6	1.503 (11)	C20—H20	0.9500
C5—H5A	0.9900	C21—C22	1.382 (11)
C5—H5B	0.9900	C21—H21	0.9500
C6—C7	1.533 (11)	C22—H22	0.9500
			
O4—Sn1—O1	82.3 (2)	H6A—C6—H6B	107.7
O4—Sn1—C5	104.7 (3)	C8—C7—C6	113.3 (8)
O1—Sn1—C5	102.1 (3)	C8—C7—H7A	108.9
O4—Sn1—C1	104.1 (3)	C6—C7—H7A	108.9
O1—Sn1—C1	108.7 (3)	C8—C7—H7B	108.9
C1—Sn1—C5	140.1 (3)	C6—C7—H7B	108.9
O4—Sn1—O2	137.44 (19)	H7A—C7—H7B	107.7
O1—Sn1—O2	55.25 (19)	C7—C8—H8A	109.5
C5—Sn1—O2	88.1 (3)	C7—C8—H8B	109.5
C1—Sn1—O2	89.0 (3)	H8A—C8—H8B	109.5
O4—Sn1—O5	53.64 (18)	C7—C8—H8C	109.5
O1—Sn1—O5	135.77 (19)	H8A—C8—H8C	109.5
C5—Sn1—O5	87.8 (3)	H8B—C8—H8C	109.5
C1—Sn1—O5	87.5 (3)	O2—C9—O1	119.5 (7)
O2—Sn1—O5	168.92 (18)	O2—C9—C10	120.8 (7)
C9—O1—Sn1	102.7 (5)	O1—C9—C10	119.6 (7)
C9—O2—Sn1	82.4 (4)	C11—C10—C15	119.7 (7)
C11—O3—H3O	120.0	C11—C10—C9	121.1 (7)
C16—O4—Sn1	106.0 (4)	C15—C10—C9	119.2 (7)
C16—O5—Sn1	81.3 (4)	O3—C11—C12	117.1 (8)
C18—O6—H6O	120.0	O3—C11—C10	122.6 (7)
C2—C1—Sn1	116.0 (5)	C12—C11—C10	120.3 (8)
C2—C1—H1A	108.3	C13—C12—C11	119.7 (9)
Sn1—C1—H1A	108.3	C13—C12—H12	120.1
C2—C1—H1B	108.3	C11—C12—H12	120.1
Sn1—C1—H1B	108.3	C12—C13—C14	121.0 (9)
H1A—C1—H1B	107.4	C12—C13—H13	119.5
C3—C2—C1	114.3 (7)	C14—C13—H13	119.5
C3—C2—H2A	108.7	C15—C14—C13	119.2 (9)
C1—C2—H2A	108.7	C15—C14—H14	120.4
C3—C2—H2B	108.7	C13—C14—H14	120.4
C1—C2—H2B	108.7	C14—C15—C10	120.2 (8)
H2A—C2—H2B	107.6	C14—C15—H15	119.9
C2—C3—C4	114.0 (9)	C10—C15—H15	119.9
C2—C3—H3A	108.8	O5—C16—O4	119.1 (6)
C4—C3—H3A	108.8	O5—C16—C17	122.5 (7)
C2—C3—H3B	108.8	O4—C16—C17	118.4 (6)
C4—C3—H3B	108.8	C22—C17—C18	119.7 (7)
H3A—C3—H3B	107.7	C22—C17—C16	120.5 (6)
C3—C4—H4A	109.5	C18—C17—C16	119.9 (6)
C3—C4—H4B	109.5	O6—C18—C19	117.5 (7)
H4A—C4—H4B	109.5	O6—C18—C17	123.8 (7)
C3—C4—H4C	109.5	C19—C18—C17	118.7 (7)
H4A—C4—H4C	109.5	C20—C19—C18	120.2 (8)
H4B—C4—H4C	109.5	C20—C19—H19	119.9
C6—C5—Sn1	111.8 (5)	C18—C19—H19	119.9
C6—C5—H5A	109.2	C19—C20—C21	121.5 (8)
Sn1—C5—H5A	109.2	C19—C20—H20	119.2
C6—C5—H5B	109.2	C21—C20—H20	119.2
Sn1—C5—H5B	109.2	C22—C21—C20	118.6 (8)
H5A—C5—H5B	107.9	C22—C21—H21	120.7
C5—C6—C7	113.4 (7)	C20—C21—H21	120.7
C5—C6—H6A	108.9	C17—C22—C21	121.3 (8)
C7—C6—H6A	108.9	C17—C22—H22	119.4
C5—C6—H6B	108.9	C21—C22—H22	119.4
C7—C6—H6B	108.9		
			
O4—Sn1—O1—C9	179.6 (5)	Sn1—O1—C9—O2	−4.1 (8)
C5—Sn1—O1—C9	−76.9 (5)	Sn1—O1—C9—C10	175.5 (5)
C1—Sn1—O1—C9	77.3 (5)	O2—C9—C10—C11	3.9 (11)
O2—Sn1—O1—C9	2.1 (4)	O1—C9—C10—C11	−175.7 (7)
O5—Sn1—O1—C9	−176.4 (4)	O2—C9—C10—C15	−178.8 (8)
O4—Sn1—O2—C9	−5.8 (6)	O1—C9—C10—C15	1.7 (11)
O1—Sn1—O2—C9	−2.1 (4)	C15—C10—C11—O3	−178.5 (9)
C5—Sn1—O2—C9	104.0 (5)	C9—C10—C11—O3	−1.2 (13)
C1—Sn1—O2—C9	−115.8 (5)	C15—C10—C11—C12	0.7 (13)
O5—Sn1—O2—C9	172.4 (9)	C9—C10—C11—C12	178.0 (8)
O1—Sn1—O4—C16	176.7 (5)	O3—C11—C12—C13	179.2 (11)
C5—Sn1—O4—C16	76.2 (5)	C10—C11—C12—C13	0.0 (17)
C1—Sn1—O4—C16	−75.8 (5)	C11—C12—C13—C14	−1(2)
O2—Sn1—O4—C16	179.8 (4)	C12—C13—C14—C15	1(2)
O5—Sn1—O4—C16	0.2 (4)	C13—C14—C15—C10	−1(2)
O4—Sn1—O5—C16	−0.2 (4)	C11—C10—C15—C14	−0.2 (15)
O1—Sn1—O5—C16	−5.1 (6)	C9—C10—C15—C14	−177.6 (10)
C5—Sn1—O5—C16	−110.3 (5)	Sn1—O5—C16—O4	0.3 (6)
C1—Sn1—O5—C16	109.4 (5)	Sn1—O5—C16—C17	−179.9 (7)
O2—Sn1—O5—C16	−178.7 (9)	Sn1—O4—C16—O5	−0.4 (8)
O4—Sn1—C1—C2	175.7 (6)	Sn1—O4—C16—C17	179.8 (5)
O1—Sn1—C1—C2	−97.9 (6)	O5—C16—C17—C22	−176.5 (7)
C5—Sn1—C1—C2	40.6 (9)	O4—C16—C17—C22	3.2 (10)
O2—Sn1—C1—C2	−45.3 (6)	O5—C16—C17—C18	2.4 (11)
O5—Sn1—C1—C2	124.2 (6)	O4—C16—C17—C18	−177.9 (7)
Sn1—C1—C2—C3	−55.4 (10)	C22—C17—C18—O6	178.7 (8)
C1—C2—C3—C4	−169.0 (9)	C16—C17—C18—O6	−0.2 (12)
O4—Sn1—C5—C6	30.8 (6)	C22—C17—C18—C19	−2.3 (11)
O1—Sn1—C5—C6	−54.3 (6)	C16—C17—C18—C19	178.8 (7)
C1—Sn1—C5—C6	165.7 (5)	O6—C18—C19—C20	−177.9 (9)
O2—Sn1—C5—C6	−108.1 (5)	C17—C18—C19—C20	3.0 (13)
O5—Sn1—C5—C6	82.2 (5)	C18—C19—C20—C21	−1.5 (15)
Sn1—C5—C6—C7	178.9 (6)	C19—C20—C21—C22	−0.7 (15)
C5—C6—C7—C8	179.3 (9)	C18—C17—C22—C21	0.1 (12)
Sn1—O2—C9—O1	3.3 (6)	C16—C17—C22—C21	179.0 (7)
Sn1—O2—C9—C10	−176.3 (7)	C20—C21—C22—C17	1.4 (13)

### Hydrogen-bond geometry (Å, °)

**Table:**

*D*—H···*A*	*D*—H	H···*A*	*D*···*A*	*D*—H···*A*
O3—H3o···O2	0.84	1.96	2.599 (9)	132
O6—H6o···O5	0.84	2.00	2.626 (8)	131
